# Tailoring surface wettability to reduce chances of infection of COVID-19 by a respiratory droplet and to improve the effectiveness of personal protection equipment

**DOI:** 10.1063/5.0020249

**Published:** 2020-08-11

**Authors:** Rajneesh Bhardwaj, Amit Agrawal

**Affiliations:** Department of Mechanical Engineering, Indian Institute of Technology Bombay, Mumbai 400076, India

## Abstract

Motivated by the fact that the drying time of respiratory droplets is related to the spread of COVID-19 [R. Bhardwaj and A. Agrawal, “Likelihood of survival of coronavirus in a respiratory droplet deposited on a solid surface,” Phys. Fluids **32**, 061704, (2020)], we analyze the drying time of droplets ejected from a COVID-19 infected subject on surfaces of personal protection equipment (PPE), such as a face mask, of different wettabilities. We report the ratio of drying time of the droplet on an ideal superhydrophobic surface (contact angle, *θ* → 180°) to an ideal hydrophilic surface (*θ* → 0°) and the ratio of the maximum to minimum drying time of the droplet on the surfaces with different contact angles. The drying time is found to be maximum if *θ* = 148°, while the aforementioned ratios are 4.6 and 4.8, respectively. These ratios are independent of the droplet initial volume, ambient temperature, relative humidity, and thermophysical properties of the droplet and water vapor. We briefly examine the change in drying time in the presence of impurities on the surface. Besides being of fundamental interest, the analysis provides insights that are useful while designing the PPE to tackle the present pandemic.

The ongoing pandemic COVID-19 caused by coronavirus has infected millions and killed hundreds of thousands of people throughout the world. A large body of ongoing research on COVID-19 is focused on understanding the mechanism of the spread of infection and to mitigate the rate of infection. One of the main mechanisms of the transmission of COVID-19 is by respiratory droplets deposited on a surface (fomite). Such droplets are shown by a schematic in Fig. 1. These droplets are ejected while coughing, sneezing, or even speaking moistly. The drying time of such droplets on a surface is particularly important since it decides the duration over which the coronavirus can get transmitted from an infected person to another person through contact with a contaminated droplet. The loss in infectivity of different viruses upon drying of droplets in which the virus was originally present has already been demonstrated experimentally.^1^ Although a previous study^2^ mentioned that the coronavirus can persist up to a few days on certain surfaces, a substantial reduction in the value of the virus titer with time was reported. Moreover, as reviewed by Kampf *et al.*,^3^ the chances of transmissibility of viruses from contaminated surfaces to hands are rather less. These authors mentioned that only 31.6% and 1.5% of viruses are transferred from the surface to the hand over a 5 s contact for influenza A and parainfluenza virus 3, respectively. Therefore, it is reasonable to assume substantially reduced chances of infection after the drying of the respiratory droplets.

**FIG. 1.**
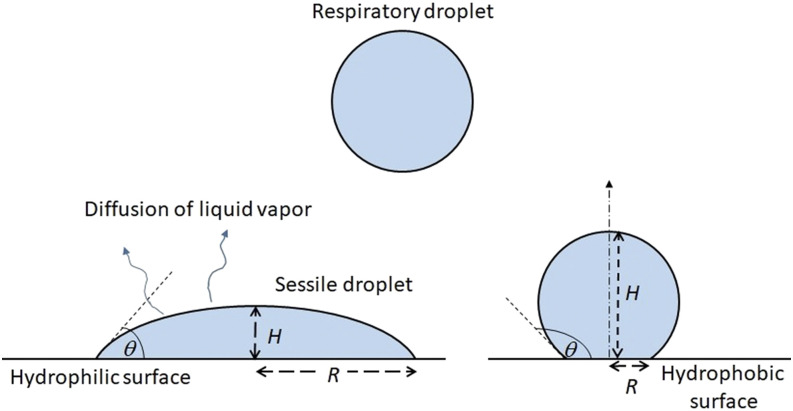
Schematic of the problem considered in the present study.

Previous studies have mapped the size of the respiratory droplets on the order of *O*(10) *μ*m to *O*(100) *μ*m. For example, Han *et al.*^4^ recorded the droplet size distribution during sneezing and reported around 360 *μ*m as the mean diameter from 44 sneezes of 20 healthy subjects. However, no study has reported the size distribution of such droplets deposited on a surface. Very recently, our earlier study^5^ brought out the importance of studying the drying time of the droplet deposited on a surface and its connection to the spread of the COVID-19 pandemic. The influence of ambient temperature, relative humidity, droplet volume, and surface wettability (contact angle) of a hydrophilic surface on the drying time was reported, and the drying time was found to correlate with the growth rate of the pandemic for six cities examined in this work.^5^

Previous studies also quantified the effect of wind speed on airborne droplets. Dbouk and Drikakis^6^ computationally showed that for a mild human cough in the air at 20 °C and 50% relative humidity, the saliva droplets can travel up to 6 m for a wind speed varying from 4 km/h to 15 km/h. In a follow-up study,^7^ the authors computationally assessed the effectiveness of the face mask for avoiding transmission of respiratory droplets passing through it. They concluded that several droplets accumulate around and away from the mask during coughing events, implying the need for social distancing to avoid the infection. Similarly, in a recent experimental study,^8^ the masks were able to significantly reduce the speed and range of the respiratory jets. However, some leakage of the cough cloud through the mask and from small gaps along the edges of the mask was reported in this study.^8^ Another recent study^9^ examined the fecal–oral transmission of the coronavirus and showed that the virus-laden droplets could be transmitted out of a toilet bowl by a flushing-induced turbulent flow.

While previous studies have examined the effectiveness of personal protection equipment (PPE)/face mask in suppressing the transmission of COVID-19 by respiratory droplets, the time during which the droplets reside on the PPE/mask and its dependency upon the contact angle remains unknown. Since a shorter drying time corresponds to lesser chances of COVID-19 infection as discussed earlier, it is worthwhile to examine the possibility of the reduction in the drying time as a function of the contact angle. Therefore, in the present study, we examine the role of surface wettability in the drying time of the droplet on various surfaces and determine the ratio of maximum to minimum drying times on different surfaces.

First, we present a model employed to estimate the drying time on a surface with a given contact angle. An aqueous respiratory droplet of volume 5 nl is considered, which is representative of a droplet produced during a cough, sneeze, or speech event.^4^ The corresponding diameter of the droplet in the air is 214 *μ*m. Droplets smaller than 100 *µ*m remain airborne, while the larger droplets being heavier settle down.^10^ The droplet is assumed to be deposited as a spherical cap on the substrate. Since the wetted diameter of the droplet is significantly smaller than the capillary length (2.7 mm for water), the droplet maintains a spherical cap shape while evaporating.^11^ The volume (*V*), contact angle (*θ*), and surface area (*A*) for a spherical cap droplet are expressed as follows:

$$V=\frac{\pi H}{6}\left(3R^{2}+H^{2}\right),\theta =2{\text{tan}}^{-1}\frac{H}{R},\;A=\pi \left(R^{2}+H^{2}\right),$$ (1)

where *H* and *R* are droplet height and wetted radius, respectively.

We consider a diffusion-limited and quasi-steady evaporation (or drying) of a sessile droplet on a partially wetted surface (Fig. 1). These assumptions for the evaporation of a nanoliter water droplet have been justified in our previous study.^5^ The droplet is assumed to be isothermal at ambient temperature. The mass loss rate (kg/s) of an evaporating sessile droplet is expressed as follows:^12,13^

$$\dot{m}=-\pi R\lambda \frac{g\left(\theta \right)}{{\left(1+\cos\theta \right)}^{2}},$$ (2)

where prefactor *λ* is given by *λ* = *D*_*wa*_*c*_*sat*_(1 − *R*_*H*_). *R*, *D*_*wa*_, *c*_*sat*_, and *R*_*H*_ are droplet wetted radius, diffusion coefficient of water vapor in air (m^2^/s), saturated concentration of water vapor (kg/m^3^), and relative humidity, respectively. The function *g*(*θ*) is defined as follows:^12,13^

$$\begin{array}{ccc} g\left(\theta \right) & = & {\left(1+\cos\theta \right)}^{2}\left[\frac{\sin\theta }{1+\cos\theta }\right.\\ & & \left.+\;4\int_{0}^{\infty }\frac{1+\cosh2\theta \tau }{\sinh2\pi \tau }\tanh\left[\left(\pi -\theta \right)\tau \right]d\tau \right]. \end{array}$$ (3)

The saturated concentration (kg/m^3^) of water vapor at a given ambient temperature (*T*) is obtained using the following fourth order polynomial, fitted using the available data^14^ (coefficient of determination, *R*^2^ ≈ 1):

$$\begin{array}{ccc} c_{sat} & = & 4.35\times 10^{-9}T^{4}-4.53\times 10^{-8}T^{3}+1.79\times 10^{-5}T^{2}\\ & & +\;2.35\times 10^{-4}T+5.07\times 10^{-3}, \end{array}$$ (4)

where *T* is the temperature in °C (0.01 °C ≤ *T* < 100 °C). The dependence of the diffusion coefficient (m^2^/s) of water vapor in air on temperature (°C) is given by^15,16^

$$D_{wa}\left(T\right)=2.5\times 10^{-4}\exp\left(-\frac{684.15}{T+273.15}\right).$$ (5)

We consider the droplet with pinned contact line throughout the evaporation, i.e., constant contact radius (CCR) mode of the evaporation. This condition is representative of real surfaces since they are usually rough or contain impurities (dust, etc.), which helps to pin the contact line. The drying time of droplet with the pinned contact line is expressed as^13^

$$t_{f}=\frac{\rho R^{2}}{\lambda }\int_{0}^{\theta }\frac{d\theta }{g\left(\theta \right)},$$ (6)

where *ρ* is the droplet density (kg/m^3^). The integrals in Eqs. (3) and (6) are numerically solved using Simpson’s rule.

We compare the drying time of the droplet deposited on a surface (*t*_*f*_) with that for an airborne droplet of the same volume (*t*_*f*,*sph*_) in our study. The latter can be obtained by using the mass loss rate of a spherical droplet^17,18^ and is expressed as

$$t_{f,sph}=\frac{\rho R_{sph}^{2}}{2\lambda },$$ (7)

where *R*_*sph*_ is the radius of the airborne droplet. Since the sessile droplet volume is considered the same as of the airborne droplet, *R*/*R*_*sph*_ can be expressed in terms *θ* after some simplification [using Eq. (1)], and therefore, *t*_*f*_/*t*_*f*,*sph*_ [using Eqs. (6) and (7)] is expressed as follows:

$$\frac{t_{f}}{t_{f,sph}}=\frac{2^{7/3}\sin^{2}\theta }{{\left(2+\cos\theta \right)}^{2/3}{\left(1-\cos\theta \right)}^{4/3}}\int_{0}^{\theta }\frac{d\theta }{g\left(\theta \right)}.$$ (8)

Equation (8) shows that the ratio *t*_*f*_/*t*_*f*,*sph*_ is only a function of contact angle *θ*. It is independent of droplet initial volume, ambient temperature, relative humidity, and thermophysical properties of the droplet and water vapor.

To avoid mathematical singularity at *θ* = 0° and 180° in Eqs. (6) and (8), the calculations using the model are instead done at *θ* = 1° and 179°, respectively. Therefore, we present results in the following range of *θ*, 1° ≤ *θ* ≤ 179°. The measured contact angles for typical surfaces reported in the literature are listed in Table I. The properties of pure water have been employed in the present calculations to determine the drying time. Since the thermophysical properties of saliva are not very different from water, the present results provide a good estimate of the evaporation time of the respiratory droplet on different surfaces.

**TABLE I.** Values of measured contact angle on surfaces of different materials. The source of data for first five surfaces was given in Ref. 5, and the contact angles on the last two surfaces were reported in Refs. 20 and 21.

Surface	Contact angle
Glass	5°–15°; 29°
Wood	62°–74°
Stainless steel	32°
Cotton	41°–62°
Smartphone screen	74°–94°
N95 mask	97°–99°
PVC-coated surface	80°–84°

To verify the fidelity of the expression of *ṁ*, the variation of normalized mass loss rate, *ṁ*_*N*_ = *ṁ*/*Rλ*, against *θ* is plotted along with the published results in Fig. 2. The comparison shows a very good match with the previous data. In addition, the plot of *g*(*θ*) in Fig. 2 verifies the published values,^13^ *g*(0°) = 16/*π*, *g*(90°) = 2, and *g*(*θ* → 180°) ≈ 0. In addition, *t*_*f*_ is compared with exact solutions available for the limiting cases of the contact angle. The exact expressions of *t*_*f*_ for *θ* → 0° and *θ* → 180° are given by^12,13,17^

$$t_{f}=\left\{\begin{array}{cc} \frac{\pi \rho R^{2}\theta }{16\lambda },\; & \text{if}\theta \to 0\\ \frac{\rho R_{sph}^{2}}{2\log\left(2\right)\lambda },\; & \text{if}\theta \to \pi , \end{array}\right.$$ (9)

where *R*_*sph*_ is the radius of the spherical droplet just touching the surface for *θ* → 180°. We consider the evaporation of a 5 nl droplet in ambient at 25° and 50% relative humidity. The values of *c*_*sat*_ and *D* are obtained from Eqs. (4) and (5), respectively. The respective drying times obtained by Eq. (9) for the limiting cases are 6.0 and 28.4 s, while the time obtained by Eq. (6) for *θ* = 1° and 179° are 6.0 and 28.1 s, respectively. The maximum difference in the computed values with respect to the values given by exact expressions is around 1%, verifying the fidelity of the expression of the drying time [Eq. (6)]. The error in drying time for other contact angles is expected to be lesser than that at these extreme values of the contact angle. Furthermore, we compare the model prediction for a measurement^16^ of the drying of a 2 *μ*l water droplet (*R* = 1.7 mm) on glass (*θ* = 29°). The drying time was measured in the ambient at *T* = 27°, *R*_*H*_ = 35%, and the contact line was pinned for the first 90% time of the total drying time. The measured drying time of the droplet is 632 s, while the model prediction for this case is 660 s, within 5% of the measured value.

**FIG. 2.**
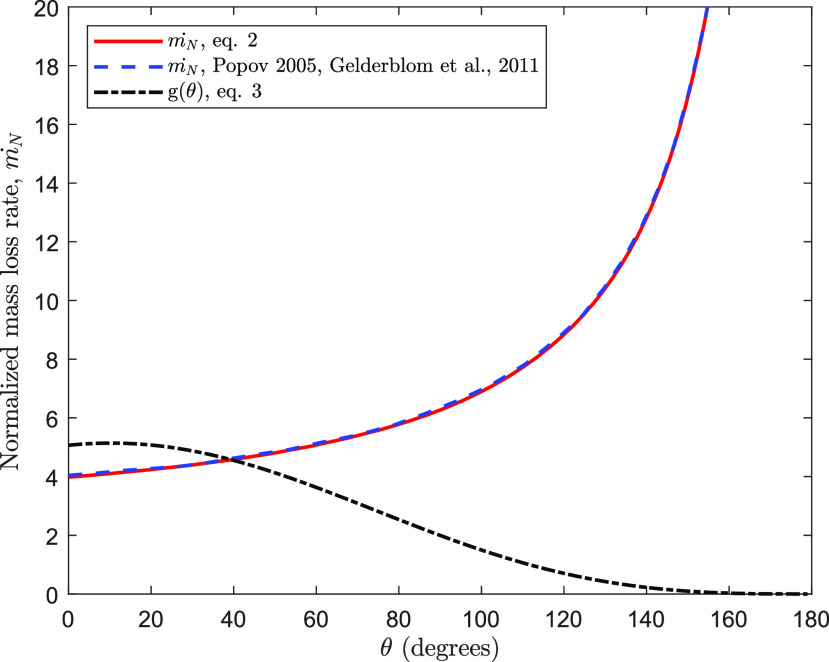
Comparison between the normalized mass loss rate (*ṁ*_*N*_ = *ṁ*/*Rλ*) as a function of contact angle (*θ*) used in the present work and that obtained by the model of Popov.^12^ The plot of *ṁ*_*N*_ for the model of Popov is reproduced by Gelderblom *et al.*^19^

Second, we present the effect of surface wettability or contact angle on the drying time (*t*_*f*_) of a 5 nl droplet for which *t*_*f*_ is plotted as a function of contact angle in Fig. 3. The variation of *t*_*f*_ with *θ* is qualitatively consistent with the published result for the CCR mode of evaporation.^13^ The drying time is maximum at *θ* = 148° (28.7 s) and minimum at *θ* = 1° (6.0 s). The plot suggests that *t*_*f*_ can be the same at two different values of the contact angle, with one of the values lying in the hydrophobic regime (121° < *θ* < 148°) and the other in the superhydrophobic regime (148° < *θ* < 180°). With an increase in *θ* beyond 148°, there is substantial lift-off of the droplet from the surface. This additional area of the liquid–gas interface, which becomes available for the diffusion of liquid vapor in the ambient, reduces *t*_*f*_ as the surface becomes superhydrophobic. We note the increase in *t*_*f*_, by about a factor of 4.8, with a change in the contact angle from 1° to 148°. However, for a larger *θ* (148° < *θ* < 180°), *t*_*f*_ varies within 2%.

**FIG. 3.**
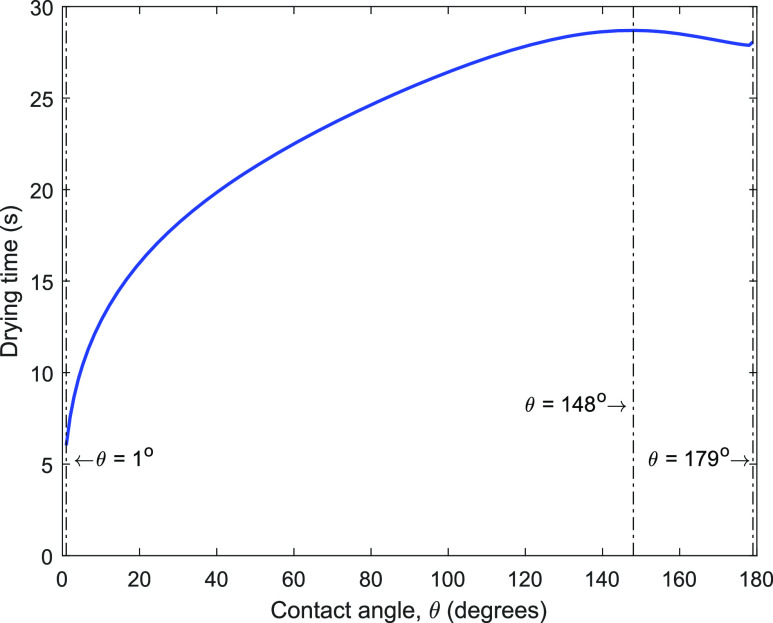
Drying time vs contact angle for a water droplet of initial volume 5 nl. These calculations are for ambient temperature of 25 °C and R_*H*_ = 50%.

The plot in Fig. 3 further suggests that to reduce *t*_*f*_ on a surface (*θ* < 148°), we can make it more hydrophilic, as expected. However, on the other hand, if the surface is superhydrophobic (*θ* > 148°) to begin with, the surface should be made even more hydrophobic for reducing the drying time further, which is somewhat counter-intuitive. The analysis further suggests that there is a limit beyond which *t*_*f*_ on a hydrophobic surface cannot be reduced; this *t*_*f*_ at *θ* = 179° is about 4.6 times larger than on a perfectly hydrophilic surface (*θ* = 1°). Therefore, to significantly reduce *t*_*f*_ on a superhydrophobic surface, the only way is to make it substantially hydrophilic. Since the *t*_*f*_ curve in Fig. 3 passes through a maximum, an incorrect amount of addition of hydrophilicity can however be counter-productive.

Figure 4 presents the ratio of the drying time of a sessile droplet on a surface (*t*_*f*_) to that of the spherical droplet suspended in air (*t*_*f*,*sph*_) of the same volume. This figure shows that the drying time for a sessile droplet is more than a freely suspended droplet other than for small contact angles (*θ* < 38.5°). This is perhaps not surprising as mass transfer from one side of the droplet is inhibited due to the presence of the solid surface, leading up to a 46% increase in the drying time for 38.5° < *θ* ≤ 148°. However, a droplet of very small contact angle has a very large wetted radius; the consequent increase in the droplet surface area compensates for the presence of the substrate surface, leading to a reduction in the drying time.

**FIG. 4.**
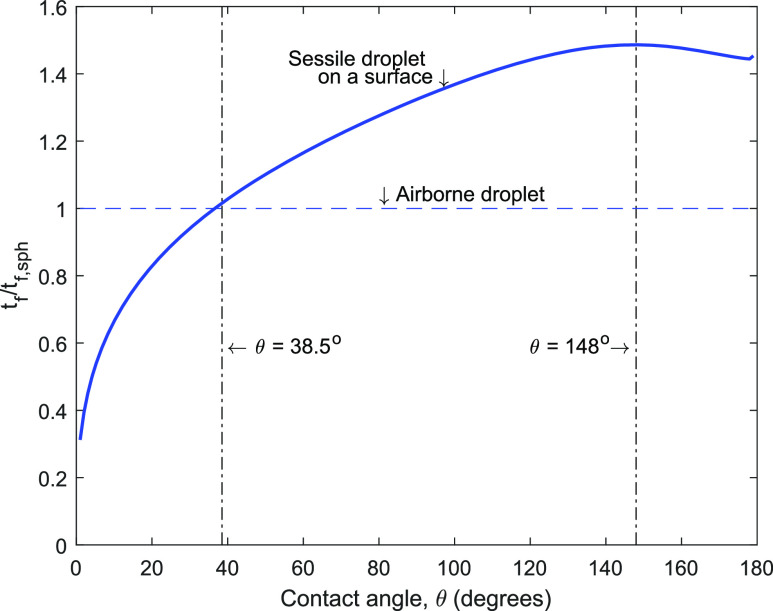
Ratio of drying time of a sessile droplet on a surface (*t*_*f*_) to that of a spherical droplet suspended in air (*t*_*f*,*sph*_) of the same volume.

To examine for the presence of universal constants in the drying time, we compute two ratios as follows. We first determine the ratio of maximum to minimum drying times for a given droplet, $t_{f}{|}_{\theta =148^{○}}/t_{f}{|}_{\theta =1^{○}}$. Figure 4 allows us to determine the ratio and it is 4.6. We next determine the ratio of drying times on an ideal hydrophobic surface (*θ* → 180°) to an ideal hydrophilic surface (*θ* → 0°). Using data in Fig. 4, this ratio, $t_{f}{|}_{\theta =179^{○}}/t_{f}{|}_{\theta =1^{○}}$, is computed as 4.8. These ratios are independent of droplet initial volume, ambient temperature, relative humidity, and thermophysical properties of the droplet and water vapor.

As an application of the universal drying curve in Fig. 4, we note that the range of droplet volume produced during coughing/sneezing/speaking is from 4 pl to 262 nl. ^4^ The respective droplet diameter is 20 *μ*m–800 *μ*m; the universal drying curve in Fig. 4 applies to this entire range of droplet size. The present calculation suggests that the drying time for respiratory droplets on a surface with *θ* = 148° varies between 0.25 s and 402 s.

Third, the effect of the presence of impurities and surface roughness is examined further. More often than not, the surface on which a droplet rests is not in a pristine state, and there are always impurities that are present. The surface is also not perfectly smooth. It has been shown that contamination on a surface increases the contact angle.^22^ Therefore, such impurities are not helpful for a surface since they would increase the contact angle and consequently the drying time (Fig. 4). Similarly, it is known that by increasing roughness, the hydrophilic surface becomes more hydrophilic, and hydrophobic becomes more hydrophobic,^11^ if the droplet is in the Wenzel state. Therefore, increasing roughness is beneficial for any given hydrophilic surface since it will help in reducing the drying time (Fig. 4) as well as the chances of infection.

Finally, we discuss the relevance of the present study to the design of the PPE/face mask that is used to avoid COVID-19 infection. In a very recent study,^20^ SEM images of a surface of an N95 mask show that the outer surface is composed of cross-linked 20 *μ*m polypropylene fibers; the measured contact angle of water droplets on this surface is around 100° (Table I). In the context of personal protection kit (PPE), the WHO recommends that PPE bodywear should be PVC-coated^23^ and the contact angle is around 82° on such a surface.^21^ In a recently proposed low-cost mask,^24^ the contact angle of the outer layer of the mask (polypropylene) is around 120°. Hence, the range of the contact angle (80°–120°) used in the PPE corresponds to a relatively long drying time on these surfaces (around 25 s–28 s for a 5 nl droplet, Fig. 3), as compared to a hydrophilic surface. As discussed earlier, a longer drying time corresponds to larger chances of the infection of COVID-19, and therefore, it is desirable to reduce the drying time.

Therefore, our study shows that by tailoring the surface wettability of the surface, drying time and, thereby, chances of the infection of COVID-19 can be reduced. As discussed earlier, making a surface more hydrophilic reduces the drying time, and therefore, it is advisable to use hydrophilic surfaces for mask/PPE and frequently touched surfaces in spaces, where the outbreak is most likely to occur (e.g., common area in hospitals). In the case of N95 mask/PPE bodywear, a reduction in contact angle to 10° (hydrophilic) reduces the chances of the infection of COVID-19 by around 38%.

There are a few limitations of the model presented here. The saliva/mucus droplets are ejected at body temperature (37 °C) and could exhibit thermocapillary convection inside the droplet (Marangoni effect) while evaporating on the surface.^25^ This could influence the drying time of the droplet. Furthermore, the ambient air is assumed to be quiescent, while air convection outside the droplet could help to reduce the drying time further. Such convection could influence the time as a function of contact angle. We did not consider the effect of solute in saliva/mucus during droplet drying. However, the change in the drying times owing to the presence of solute is expected to be minor.

In closure, this study examines the drying time of a droplet on different surfaces, which has implications in reducing the chances of the infection of COVID-19 by a respiratory droplet deposited on a surface. We find that the drying time increases rapidly until the contact angle of *θ* = 148°, beyond which the drying time is less sensitive to the surface on which the droplet is placed. We also find that to reduce the drying time and the chances of the infection of COVID-19, a hydrophilic surface should be employed for mask/PPE and frequently touched surfaces. The normalized drying time is independent of the thermophysical properties of the droplet and initial volume, and therefore, the results computed for a water droplet are equally applicable to respiratory droplets and droplets of other fluids. These insights can help design better masks, and we suggest roughening of the mask/PPE surface and frequently touched surfaces to reduce the drying time of a droplet as well as the chances of the infection of COVID-19 by a respiratory droplet ejected by an infected person.

## DATA AVAILABILITY

The data that support the findings of this study are available from the corresponding author upon reasonable request.
